# Solitary Nodule in the Hard Palate

**DOI:** 10.1111/odi.70192

**Published:** 2026-05-12

**Authors:** Sara Lia Gonçalves de Lima, Poliana Soares Cruz, Maria Gabriela Candido Dias, Láysa Tavares de Melo, Laura Maria dos Santos Reis Rocha de Castro, Felipe Paiva Fonseca, Brunno Santos de Freitas Silva

**Affiliations:** ^1^ Center for Research in Oral Systemic Health, School of Dentistry Federal University of Goias Goiania Brazil; ^2^ School of Dentistry Integrated Colleges of South America Caldas Novas Brazil; ^3^ Federal University of Uberlândia Uberlândia Brazil; ^4^ Department of Oral Surgery and Pathology, School of Dentistry Universidade Federal de Minas Gerais Belo Horizonte Brazil; ^5^ Department of Stomatologic Sciences, School of Dentistry Federal University of Goiás Goiânia Brazil; ^6^ University of Anápolis Anápolis Brazil

**Keywords:** diagnosis, immunohistochemistry, neurofibroma

## 1 Case Report

A 48‐year‐old mixed‐race female patient presented for evaluation of a painless swelling, describing it as a “lump on the roof of the mouth” that had been slowly enlarging over approximately 2 years. She reported no pain, bleeding, or systemic symptoms. Intraoral examination revealed a well‐circumscribed, soft, non‐tender nodule approximately 1 cm in diameter, with a smooth surface, mucosa‐like color, and a central ulceration. The lesion was located on the hard palate, adjacent to teeth 24 and 25 (Figure 1). The surrounding mucosa appeared normal, and there was no evidence of tooth mobility or underlying bone involvement.

**FIGURE 1:**
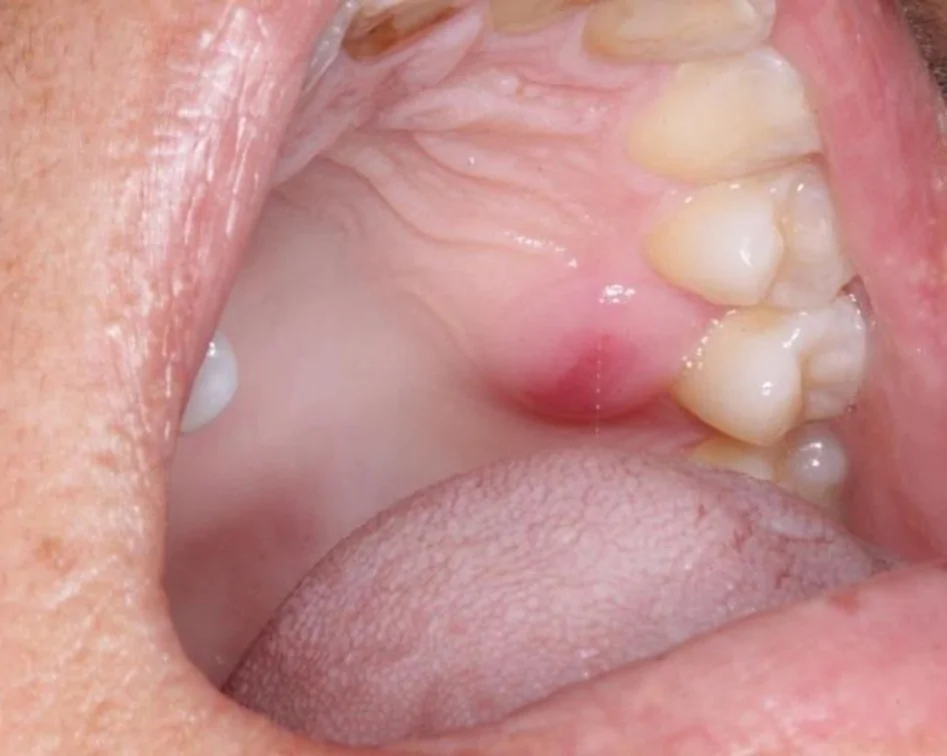
Clinical presentation of the lesion on the hard palate: a well‐circumscribed, soft nodule with mucosa‐like coloration and central ulceration, located adjacent to teeth 24 and 25.

## 2 What Is Your Diagnosis?

Based on the patient's history and physical examinations, what is the likely diagnosis?

- Pleomorphic adenoma
- Peripheral odontogenic fibroma
- Traumatic fibroma
- Neurofibroma

## 3 Diagnosis

Answer D: Neurofibroma. After incisional biopsy, histopathological examination revealed a proliferation of spindle‐shaped cells with wavy nuclei or ovoid cells, embedded in a myxoid stroma. Immunohistochemical analysis showed strong positivity for S‐100 protein, Ki‐67 positivity of less than 5%, and negativity for smooth muscle actin (SMA), CD34, H‐caldesmon, and desmin, confirming the diagnosis of neurofibroma (Figure 2).

**FIGURE 2:**
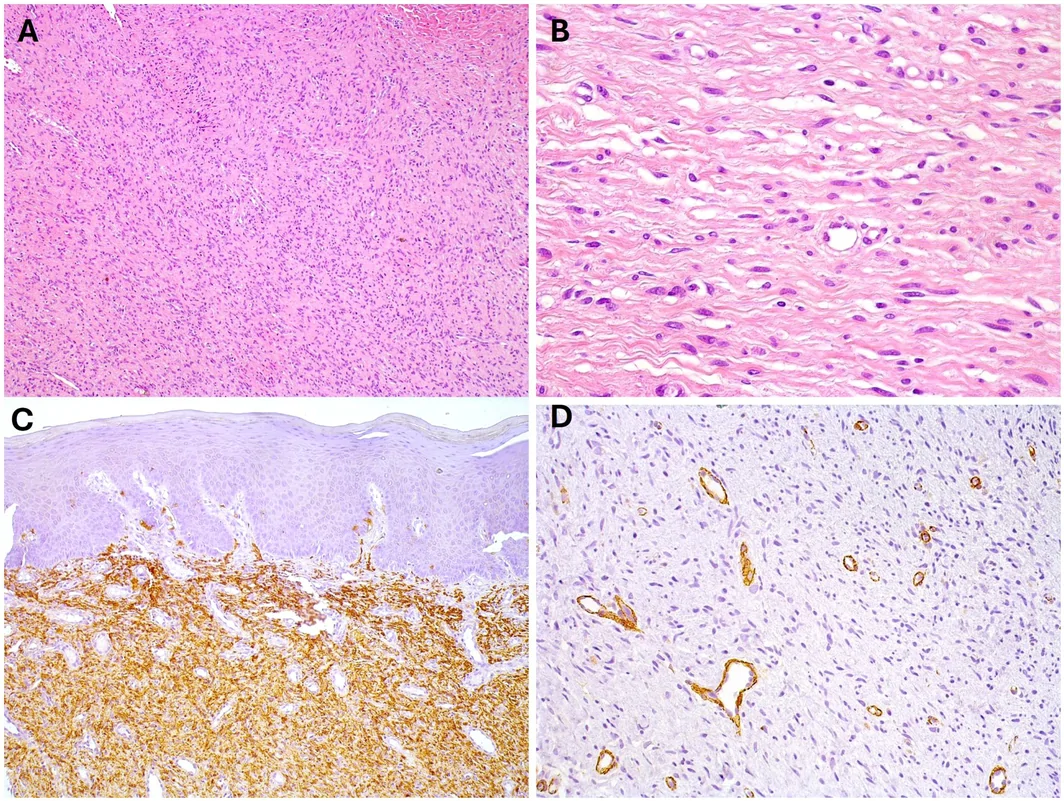
Histopathological and immunohistochemical analysis. Photomicrographs showing: (A) proliferation of spindle‐shaped cells and (B) wavy nuclei within a myxoid stroma (Hematoxylin and Eosin staining, 10× and 40×, respectively); (C) strong immunoreactivity for S‐100 protein in tumor cells (10×); and (D) absence of Smooth Muscle Actin (SMA) expression in tumor cells (20×).

Neurofibroma is a benign peripheral nerve sheath tumor composed of Schwann cells, fibroblasts, and perineurial‐like cells. Its occurrence in the oral cavity is rare, accounting for approximately 6.5% of cases. Among intraoral sites, the tongue is most frequently affected, followed by the buccal mucosa, gingiva, and more rarely, the hard palate (Narasimhan et al. 2024; Guo et al. 2024; Taketomi et al. 2021; Bharath et al. 2014).

In the present case, the lesion presented as a slow‐growing, soft nodule with mucosa‐like coloration and central ulceration. Although ulceration is not characteristic of benign neural tumors, it may occur secondary to chronic trauma or superficial ischemia. Malignant salivary gland tumors, such as mucoepidermoid carcinoma and adenoid cystic carcinoma, can also present with ulceration, highlighting the importance of a thorough differential diagnosis. Previous reports emphasize that palatal neurofibromas can be clinically mistaken for salivary gland tumors or other benign neoplasms, underscoring the critical role of histopathological and immunohistochemical analysis (Angiero et al. 2016; Wu et al. 2016; Taketomi et al. 2021).

Histologically, neurofibromas are composed of non‐encapsulated spindle cells with wavy nuclei distributed in a collagenous or myxoid stroma. In our case, immunohistochemical analysis showed strong positivity for S‐100 protein. This immunoprofile, consistently reported in the literature, is crucial for diagnosis, confirming the neural origin and excluding myoepithelial, myogenic, and malignant tumors (Thompson et al. 2020; Taketomi et al. 2021; Guo et al. 2024).

Although plexiform neurofibromas are frequently associated with neurofibromatosis type 1 (NF1), solitary neurofibromas can occur as isolated, non‐syndromic lesions.

While the association between NF1 and oral neurofibromas is not mandatory, it is well documented and should always be considered when clinical features are suggestive (Shekar et al. 2015; Wu et al. 2016; Kınış et al. 2013). Oral manifestations can involve soft tissues or, more rarely, bone—with a predilection for the mandible, although palatal and maxillary involvement have also been reported (Sharma et al. 2023; Guo et al. 2024).

Neurofibromas of the hard palate are extremely rare and may pose diagnostic challenges. Histopathological and immunohistochemical confirmation are essential for accurate diagnosis and appropriate clinical management.

## 4 Outcome

The patient was referred for clinical evaluation by a neurologist to investigate the possibility of neurofibromatosis. Complete surgical excision of the lesion is planned.

## Author Contributions

**Sara Lia Gonçalves de Lima:** writing – review and editing, conceptualization, investigation. **Poliana Soares Cruz:** conceptualization, writing – review and editing. **Maria Gabriela Candido Dias:** conceptualization, formal analysis. **Láysa Tavares de Melo:** conceptualization, formal analysis. **Laura Maria dos Santos Reis Rocha de Castro:** conceptualization, writing – review and editing. **Felipe Paiva Fonseca:** methodology, writing – review and editing. **Brunno Santos de Freitas Silva:** conceptualization, writing – review and editing, writing – original draft.

## Consent

The patient reported in this manuscript provided written informed consent for the publication of the case details.

## Conflicts of Interest

The authors declare no conflicts of interest.

## Data Availability

The data that support the findings of this study are available on request from the corresponding author. The data are not publicly available due to privacy or ethical restrictions.
